# Laugier–Hunziker Syndrome: A Rare Cause of Oral and Acral Pigmentation

**DOI:** 10.4103/0974-2077.79199

**Published:** 2011

**Authors:** Silonie Sachdeva, Shabina Sachdeva, Pranav Kapoor

**Affiliations:** *Carolena Skin, Laser & Research Centre, Jalandhar, Punjab, India*; 1 *Faculty of Dentistry, Jamia Millia Islamia, New Delhi, India*

**Keywords:** Benign, longitudinal melanonychia, oral pigmentation

## Abstract

Laugier–Hunziker syndrome (LHS) is an acquired, benign pigmentary skin condition involving oral cavity including lower lip in the form of brown black macules 1–5 mm in size, frequently associated with longitudinal melanonychia. There is no underlying systemic abnormality or malignant predisposition associated with LHS, and therefore the prognosis is good. Important differential diagnoses include Peutz Jeghers syndrome and Addison’s disease among other causes of oral and acral pigmentation. Treatment is sought mainly for cosmetic reasons and Q-switched Nd-Yag laser/ Q-switched alexandrite therapy and cryosurgery have been tried with varying success.

## INTRODUCTION

Laugier–Hunziker syndrome (LHS) is an idiopathic macular hyperpigmentation of skin characterized by brownish black spots on oral mucosa including lips associated with longitudinal melanonychia of nails. Extended mucocutaneous pigmentation has been seen in few cases on the neck, thorax, abdomen, dorsal and lateral aspects of the fingers, palms and soles, and the perineum.

## CASE REPORT

A 50-year-female presented with history of pigmented spots on lower lip, fingers, and toes which had developed gradually over last 2 years. Patient also complained of pigmentation on the tongue which had developed in last 6 months. There was no history of pain or burning sensation in oral cavity. There was no history of any drug intake (including antimalarials, minocycline or gold therapy), exposure to radiation, PUVA, or any trauma prior to the onset of the pigmentation. There was no history of precocious puberty in the patient or history of similar pigmentation in the early childhood or young age. There was no history of abdominal pain, diarrhea, vomiting, rectal bleeding, hematemesis, fatigue, hypotension or weight loss associated with the onset of pigmentation. There was no family history of similar skin disease. Clinical intra-oral examination of the oral cavity revealed moderate orodental hygiene. There were brown colored pigmented macules 1–3 mm in size on lower lip [Figure 1a]. Upper lip was spared. There was slate-gray pigmentation of the tongue [Figure 1b]. There was no pigmentation of the buccal mucosa, soft palate, hard palate, or gingiva. There was no ulceration inside the mouth. Examination of hands revealed round to oval well-defined brown-colored macules 1-5 mm in diameter on the palmar aspect of the tips of all the fingers [Figure 1c]. There was no darkening of the palmar creases. Similar pigmentation was seen on tips of all the toes [Figure 1d]. The dorsal surface of palms and soles also had few pigmented patches. The thumb nails showed longitudinal brownish black vertical bands 5-7 mm thick in size [Figure 2]. There was no periungual extension of the brown-black pigmentation. General physical and systemic examination was normal. Laboratory investigations showed a hemoglobin value of 11.2 gm%. ESR was raised to 42 mm/h. Ultrasound of abdomen was normal. A skin biopsy was taken from the macule on the left thumb, which revealed marked hyperkeratosis and acanthosis and increased amount of melanin in the basal layer of the epidermis [Figure 3]. There was no increase in the number of melanocytes and no junctional activity or melanin incontinence. The patient was advised to undergo endoscopy of the gastrointestinal tract, which came out to be normal. Patient was diagnosed as a classical case of LHS, which is a rare cause of oral and acral pigmentation.

**Figure 1:**
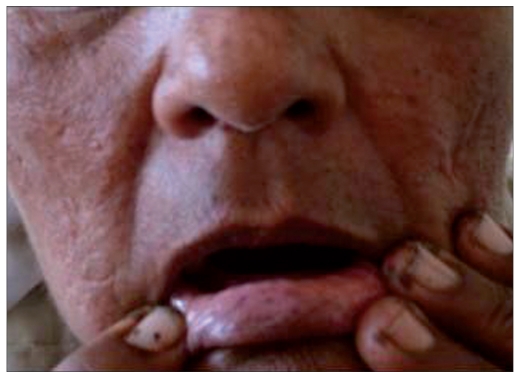
(a) Pigmentation on the lower lip

**Figure 1:**
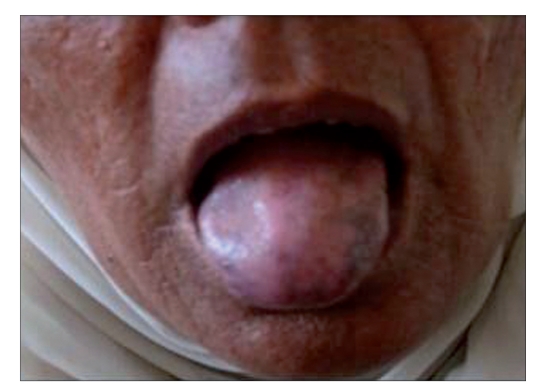
(b) Pigmentation on the tongue

**Figure 1:**
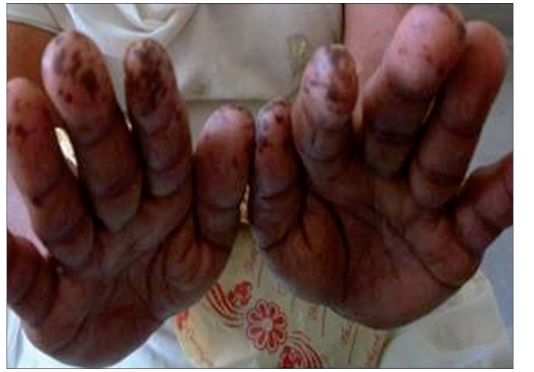
(c) brownish – black pigmentation on tips of fingers

**Figure 1:**
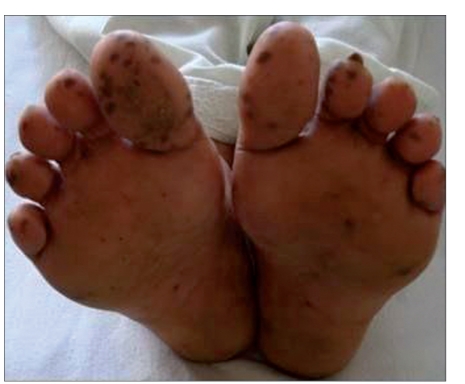
(d) brownish – black pigmentation on tips of toes

**Figure 2:**
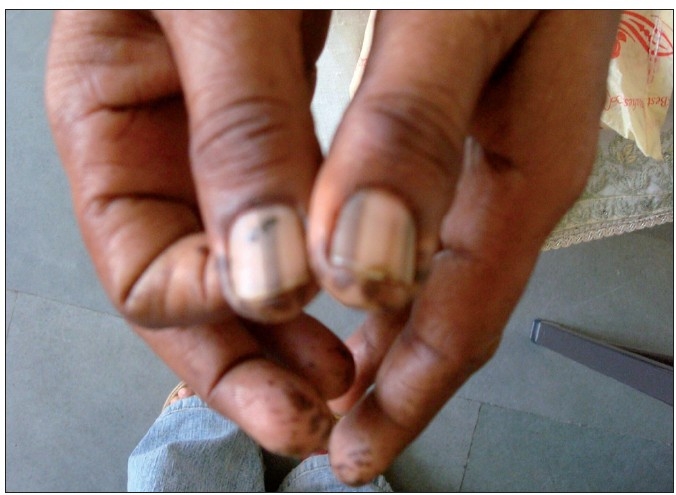
Longitudinal melanonychia of thumb nails

**Figure 3:**
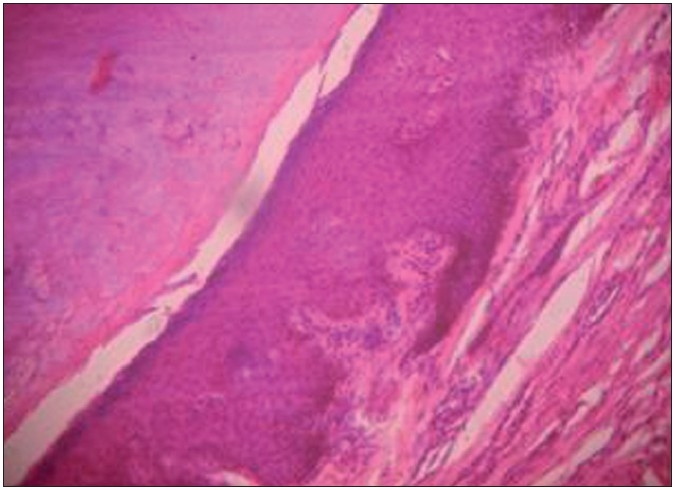
Biopsy of a pigmented macule reveals an increase in melanin in the basal layer of the epidermis (H and E, ×100)“

## DISCUSSION

LHS was described initially in 1970 as an acquired, benign skin condition characterized by hyperpigmented macules on the lips and buccal mucosa associated with longitudinal melanonychia of nails.[1] Considered a diagnosis of exclusion and primarily reported from European countries in white population, sporadic cases have been reported from Asia including India in last decade.[2–4] The pigmentation typically develops during early to middle adulthood. A mean age of 52 years and a median age of 42 years have been reported. Oral hyperpigmentation may be the only presenting sign or may coexist with skin and nail pigmentation. The buccal mucosa and the lips (usually the lower lip) are the most commonly involved sites, but gingiva, tongue, soft palate, and the hard palate can also be involved. The pigmentation is in the form of smooth-surfaced brown-, black-, or slate-colored macules measuring 1-5 mm in size. Nail pigmentation is in the form of thick vertical longitudinal bands. One or two vertical bands may appear or nails may present with half or complete nail pigmentation. Pseudo-Hutchinsons sign when hyperpigmentation of the nail bed and matrix reflects through the transparent nailfolds simulating Hutchinson’s sign, a marker of sub-ungual melanoma, has also been reported in few cases of LHS.

When LHS is associated with nonclassic body locations or atypical features, the name idiopathic lenticular mucocutaneous hyperpigmentation has been used.[5] So far, only one case of familial LHS has been described, which involved a mother and two daughters.[6] Most reports of the histopathological changes in pigmented macules of LHS describe increased basal layer pigmentation in skin and mucosal lesions, but normal number and morphologic appearance of melanocytes. Important differential diagnosis of LHS include Peutz Jeghers syndrome (PJS) characterized by pigmentation around nose and mouth, oral cavity, palms, soles, and associated with hamartomatous gastrointestinal polyposis, which carries a high risk of malignancy.[7] Addison’s disease, which is characterized by hyperpigmentation of the skin in areas subject to increased pressure, such as over the knuckles or the skin creases and in mucous membranes, is also an important differential diagnosis of LHS. Associated features include decreased pubic and axillary hair in women, dehydration, hypotension, and abdominal pain.[8]

Less common differentials of LHS include McCune-Albright syndrome, LEOPARD syndrome, Gardener syndrome, Cronkhite-Canada syndrome, LAMB syndrome, drug-induced hyperpigmentation, friction-induced longitudinal melanonychia of the toenails, and heavy metal exposure.

Treatment is sought mainly for cosmetic reasons and include Q-switched Nd: YAG or Q-switched alexandrite laser therapy for bothersome melanosis on the skin.[9] Sun protection is important to prevent reoccurrence. Cryosurgery has also been tried for pigmentation in LHS with good results.[10]

Our patient had characteristic features of LHS. Patient was offered Q-switched Nd-Yag laser treatment for pigmentation, but she had no cosmetic concerns and denied treatment. Patient was reassured of the benign nature of the disease. The prognosis of the disease is good as there is no systemic illness or malignancy associated with LHS.

The case is being presented for its classical presentation and to reinforce the earlier observation made by Indian authors that LHS is not restricted to any particular region of the world and to reinforce the ubiquity of Laugier-Hunziker pigmentation.
